# The utility of website-based quality improvement tools for health professionals: a systematic review

**DOI:** 10.1093/intqhc/mzae068

**Published:** 2024-07-10

**Authors:** Georgie Tran, Bridget Kelly, Megan Hammersley, Jennifer Norman, Anthony Okely

**Affiliations:** Early Start, Faculty of the Arts, Social Sciences and Humanities, University of Wollongong, Wollongong, NSW 2522, Australia; Early Start, Faculty of the Arts, Social Sciences and Humanities, University of Wollongong, Wollongong, NSW 2522, Australia; Early Start, Faculty of the Arts, Social Sciences and Humanities, University of Wollongong, Wollongong, NSW 2522, Australia; Health Promotion Service, Illawarra Shoalhaven Local Health District, Warrawong, NSW 2502, Australia; Early Start, Faculty of the Arts, Social Sciences and Humanities, University of Wollongong, Wollongong, NSW 2522, Australia

**Keywords:** health, quality improvement, web tools, digital tools, narrative review

## Abstract

As technology continues to advance, it is important to understand how website-based tools can support quality improvement. Website-based tools refer to resources such as toolkits that users can access and use autonomously through a dedicated website. This review examined how website-based tools can support healthcare professionals with quality improvement, including the optimal processes used to develop tools and the elements of an effective tool. A systematic search of seven databases was conducted to include articles published between January 2012 and January 2024. Articles were included if they were peer reviewed, written in English, based in health settings, and reported the development or evaluation of a quality improvement website-based tool for professionals. A narrative synthesis was conducted using NVivo. Risk of bias was assessed using the Mixed Methods Appraisal Tool. All papers were independently screened and coded by two authors using a six-phase conceptual framework by Braun and Clarke. Eighteen studies met the inclusion criteria. Themes identified were tool development processes, quality improvement mechanisms and barriers and facilitators to tool usage. Digitalizing existing quality improvement processes (*n* = 7), identifying gaps in practice (*n* = 6), and contributing to professional development (*n* = 3) were common quality improvement aims. Tools were associated with the reported enhancement of accuracy and efficiency in clinical tasks, improvement in adherence to guidelines, facilitation of reflective practice, and provision of tailored feedback for continuous quality improvement. Common features were educational resources (*n* = 7) and assisting the user to assess current practices against standards/recommendations (*n* = 6), which supported professionals in achieving better clinical outcomes, increased professional satisfaction and streamlined workflow in various settings. Studies reported facilitators to tool usage including relevance to practice, accessibility, and facilitating multidisciplinary action, making these tools practical and time-efficient for healthcare. However, barriers such as being time consuming, irrelevant to practice, difficult to use, and lack of organizational engagement were reported. Almost all tools were co-developed with stakeholders. The co-design approaches varied, reflecting different levels of stakeholder engagement and adoption of co-design methodologies. It is noted that the quality of included studies was low. These findings offer valuable insights for future development of quality improvement website-based tools in healthcare. Recommendations include ensuring tools are co-developed with healthcare professionals, focusing on practical usability and addressing common barriers to enhance engagement and effectiveness in improving healthcare quality. Randomized controlled trials are warranted to provide objective evidence of tool efficacy.

## INTRODUCTION

Quality improvement (QI) involves stakeholders devising plans to enhance current practices to improve outcomes [1]. It is an ongoing process to continually improve practices and is often required as part of accreditation [2]. A range of frameworks are available to guide the approach to QI. For example, the ‘Plan-Do-Study-Act’ framework allows health departments to systematically compare current practices against established standards and to modify practices for continuous QI [2]. In addition, there are QI tools available. QI tools are specifically referring to instruments, such as checklists and process maps, used to implement and measure improvement initiatives, whereas QI frameworks provide systematic approaches for guiding the overall process of QI [3].

Technological advances have allowed tools to be integrated with online platforms. Research exploring how technology can support QI in a hospital setting concluded that such technology allowed for the provision of efficient and adequate feedback of performance [4]. For example, electronic health records integrated with real-time data analytics enable clinicians to receive immediate feedback on patient outcomes, facilitating prompt adjustments to care protocols and improving overall patient safety [5]. In addition, websites provide several advantages over other types of online platforms, such as more flexibility, accessibility and cross-platform compatibility [6].

As technology continues to advance and digitalization becomes more common, it is important to understand how digital tools can support QI in healthcare. There is abundant literature on QI frameworks [7–9]; however, to our knowledge, there is no literature summarizing QI tool design processes or components that can be used to guide tool development. With the advancement of technology, many digital tools will continue to be developed. QI projects can be complex and websites are a good technological solution to assist in these processes.

This systematic review aims to understand how website-based tools can support health professionals with QI. The primary research question is: What are the optimal website-based QI tool design processes and elements of an effective QI tool?

## METHODS

### Protocol and registration

This review adheres to the Preferred Reporting Items for Systematic review and meta-Analysis (PRISMA) statement [10] and prospectively registered with PROSPERO no. CRD42023451346. The completed PRISMA checklist is provided in supplementary file 1.

Institutional ethics board approval was not required for this study since it was a systematic review and did not involve human subjects as research participants.

### Eligibility criteria

Papers were included if they were peer reviewed, written in English, available in full text, based in a health setting and reported the development or evaluation of a website-based QI tool used by health professionals.

In this study, website-based tools were defined as resources such as toolkits that users can access and use autonomously through a dedicated website. Studies that examined tools on other platforms, e.g. mobile applications (tools specifically designed as a mobile application, as these are different to websites that are also mobile-friendly) were excluded. This exclusion was due to the distinct nature of mobile applications which typically depend on the hardware and operating systems of mobile devices and require more platform-specific programming, potentially affecting the consistency and generalizability of results [11].

### Data sources and search strategy

A computerized search was conducted in December 2022 and repeated in January 2024 using PubMed, MEDLINE, ScienceDirect, Wiley, Scopus, ProQuest, Education Resource Complete, and A+ Education. Published research in health information and technology emerged in 2008 but began increasing from 2012 [12]. Therefore, the databases were searched from January 2012 to January 2024. Search terms used were quality improvement tool OR quality tool AND web-based OR website-based. The complete search strategy is outlined in Supplementary File 2. Reference lists were also screened.

Identified articles were uploaded and duplicates removed in Covidence [13]. Titles and abstracts were reviewed by one author (G.T.). All potentially relevant full-text articles were independently assessed by two authors (among G.T., M.H., and J.N.). Any differences were discussed and resolved between reviewers.

### Data extraction

Data extracted by one author (G.T.) via Covidence included title, author, location, date, discipline area, study design, and study population. Two authors (G.T. and M.H.) independently extracted data on tool development process, QI mechanism, tool features/resources, and outcome measures and assessments. Differences were discussed and resolved between reviewers.

Tool development data specifically examined four key steps—research/literature review, use of theoretical framework to guide development, co-developing with stakeholders, and field testing. These steps were based on the UK Medical Research Council framework for developing and evaluating complex interventions [14].

### Data synthesis

All data were entered into Covidence and narratively synthesized on tool development processes, elements of an effective QI tool, and outcome measures and assessments. Studies could contribute information on one or more of these aspects. Thematic analysis using NVivo [15] followed a six-phase process [16]. All papers were independently coded by two authors and cross-checked for any discrepancies. A meta-analysis was not possible as there was a high degree of heterogeneity.

### Quality assessment

The Mixed Methods Appraisal Tool version 2018 [17] was used. The risk of bias was assessed independently by two authors (G.T. and M.H.). Any differences were discussed and resolved between the authors. The GRADE framework [18] was used to assess the overall body of evidence.

## RESULTS

**Figure 1:**
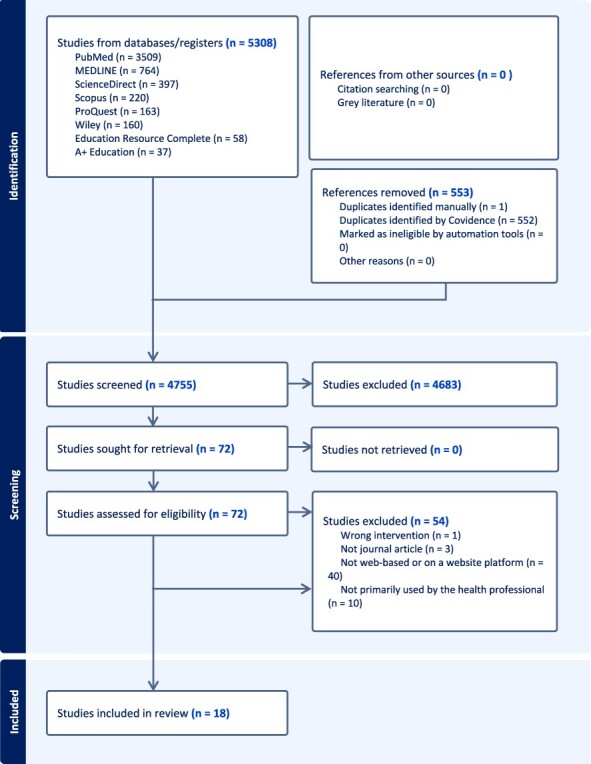
Study flow diagram of search results and the selection process.

A total of 5308 articles were screened and 18 studies met the inclusion criteria (Fig. 1).

Table 1 summarizes the study characteristics. Studies that only described tool development included qualitative studies (*n* = 3) and a quantitative descriptive study (*n* = 1). Studies that reported tool evaluation included qualitative studies (*n* = 6), quantitative descriptive studies (*n* = 2), feasibility studies (*n* = 5), and a non-randomized experimental study (*n *= 1). No studies were randomized controlled trials.

**Table 1.** Characteristics of included studies.

Title	Author, location, date	Study design	Study population (type of professional)
The endoscopy Global Rating Scale–Canada: development and implementation of a quality improvement tool [19]	MacIntosh, Canada, 2013	Qualitative study	Endoscopy facility staff
The Residency Performance Index: an Effort at Residency Quality Assessment and Improvement in Family Medicine [20]	Hoekzema, USA, 2014	Qualitative study	Family medicine programme directors
Development of a Global Rating Scale for Inflammatory Bowel Disease [21]	Bitton, Canada, 2020	Quantitative descriptive study	Inflammatory Bowel Diseases health professionals
Development and pilot study of the Primary Care Practice Improvement Tool (PC-PIT): an innovative approach [23]	Crossland, Australia, 2014	Feasibility study	GP staff
EQUSUM: Endometriosis QUality and grading instrument for SUrgical performance: proof of concept study [28]	Metzemaekers, The Netherlands, 2020	Quantitative descriptive study	Experts in endometriosis surgery
Piloting online self-audit of methadone treatment in Irish general practice: results, reflections, and educational outcomes [31]	Van Hout, UK, 2018	Qualitative study	General practitioners
Development of an Online Toolkit for Measuring Performance in Health Emergency Response Exercises [49]	Agboola, USA, 2015	Qualitative study	Health emergency response exercise planners
Improving quality in pastoral care using the Pastoral Care Activity Tracker (PCAT): a feasibility study of a digital tool within an Australian healthcare organization [29]	Calder, Australia, 2022	Feasibility study	Pastoral care coordinators
A Qualitative Evaluation of Web-Based Cancer Care Quality Improvement Toolkit Use in the Veterans Health Administration [25]	Bowman, USA, 2015	Qualitative study	Healthcare professionals
Quality improvement in preoperative assessment by implementation of an electronic decision support tool [32]	Flamm, Austria, 2013	Non-randomized experimental study	Physicians
Implementation of a Point-of-Care Radiologist-Technologist Communication Tool in a Quality Assurance Program [50]	Ong, USA, 2017	Quantitative descriptive study	Radiology staff
Safety Profile Assessment: An online tool to gauge safety-critical performance in radiation oncology [24]	Dunscombe, USA, 2015	Feasibility study	Clinical staff in radiation oncology
The use of an online comment system in clinical ethics consultation [51]	Hauschildt, USA, 2017	Qualitative study	Clinical ethics consultants
Quality Innovation Networks Share Varied Resources for Nursing Homes on Mostly User-Friendly Websites [33]	Quigley, USA, 2019	Qualitative study	Nursing home staff
Delivering Evidence-Based Online Concussion Education to Medical and Healthcare Professionals: The Concussion Awareness Training Tool (CATT) [52]	Babul, Canada, 2020	Qualitative study	Health professionals
Paediatric Endoscopy Global Rating Scale: Development of a Quality Improvement Tool and Results of a National Pilot [22]	Narula, UK, 2019	Qualitative study	Staff from paediatric endoscopy services
A Web-Based Tool to Report Adverse Drug Reactions by Community Pharmacists in Australia [30]	Fossouo Tagne, Australia, 2023	Feasibility study	Community pharmacists
Designing and developing a digital equity dashboard for the emergency department [53]	Yi, USA, 2023	Feasibility study	Emergency clinicians

Four studies only described the tool development; therefore, outcome measures were not reported [19–22]. The outcomes of the remaining 14 studies are summarized in Table 2. All 14 studies reported positive outcomes. Outcome measures varied across studies as they were dependent on the tool’s purpose. The various purposes of tools included improving clinical practice and compliance (*n* = 5), enhancing usability of traditional, paper-based or manual methods (*n* = 4), and supporting quality assurance and reflective practice (*n* = 5).

**Table 2.** Summary of tool outcome measures and assessment methods.

Author, date, location	Study population (type of professional)	Outcome measures	Outcome assessment methods	Key results
Crossland 2014, Australia [23]	GP staff	Readability, content validity and staff perceptions/process validity of the tool	Readability was assessed using the Flesch–Kincaid. Readability Formula and Gunning–Fog Index in a combined online test. Readability, content validity and process validity were assessed using a series of Likert scale questions.	Some definitions were regarded as complex and many staff (except practice managers) found element descriptions hard to understand. Required reading age of >20 years. Governance and performance were particularly difficult to understand; 19/28 staff regarded the tool as useful for assessing practice organization and function and 21 preferred it to a paper form. Eight stated that they did not think that it was usable in its current format.
Metzemaekers 2020, The Netherlands [28]	Experts in endometriosis surgery	Classification accuracy of deep endometriosis scores and stages and usability	Correct classification of the hypothetical case and the System Usability Scale.	The web-based tool improved the classification of endometriosis using the revised American Society for Reproductive Medicine (rASRM), Enzian and Endometriosis Fertility Index (EFI) stages/scores compared to the currently used paper method. The usability of the web-based tool was also superior to paper.
Van Hout 2018, UK [31]	General practitioners	Design and development, implementation and GP perspectives of the tool	Design and development (description). Implementation (quantitative chart review). GP perspectives (qualitative).	There was a high level of compliance with the Methadone Treatment Protocol guidelines by the participating GPs. The survey was well received and it was stated that it encouraged reflective practice. Some suggestions for improvement were provided. It is recognized that there are many complexities in providing treatment and care and that it would be useful to explore this through a socio-cultural lens. It is proposed that this audit process could be modified for the treatment of other chronic diseases in GP.
Agboola 2015, USA [49]	Health emergency response exercise planners from public health and healthcare agencies	Usefulness of online toolkit	Qualitative evaluation of the usefulness of the toolkit, the evaluation tool (generated form) and associated data collected.	The toolkit is demonstrated to be user-friendly and a high level of acceptability from users. It is closely aligned with relevant health emergency response capabilities and also had potential use beyond evaluation of exercises (such as the planning and execution of exercises).
Calder 2022, Australia [29]	Pastoral care coordinators	Feasibility of digital tool to record pastoral care activities	Digital activity data and user feedback surveys (6-point scale questions on perceived convenience, perceived difficulty, perceived time requirement, preference, perceived helpfulness, open feedback).	The digital tool was found to be feasible and most Pastoral Care Coordinators preferred it to paper-based methods and found it was easier to use for reporting. It was generally perceived that the data capture was improved.
Bowman 2015, USA [25]	Healthcare professionals or providers of cancer care	Characteristics of toolkits, the target users, and facilities that influenced access and use of the toolkits, and to determine whether the resources were beneficial for the users.	Semi-structured telephone interviews on individual characteristics, organizational context, and intervention characteristics affecting the use of the toolkits.	The online Toolkit series was well received by healthcare staff across different cancer specialties. The toolkits can build on established QI systems and collaborative learning networks supported the awareness of the toolkits. However, local challenges were identified, such as approval processes. Harnessing facilitators, such as the use of champions could improve implementation.
Flamm 2013, Austria [32]	Physicians	Unnecessary preoperative testing Guideline adherence in preoperative assessment Cost reduction	Tool data on six tests most frequently performed (full blood count, liver function, coagulation parameters, electrolytes, ECG, and chest X-ray) to determine tests performed without indication. Percentage of patients tested unnecessarily. Percentage of patients not tested although testing was -recommended. Percentage of patients assessed in complete accordance with guidelines. Average costs due to unnecessary tests per patient were calculated for both groups based on health insurance reimbursement rates for tests. Cost savings then calculated on six most common preoperative tests.	The number of tests per patient that were performed without indication was 3.39 ± 1.44 in the control group and 0.60 ± 1.06 in the intervention group; 97.8% of patients in the control group received at least one unnecessary test compared to 31.5% of patients in the intervention group. In the average of the six tests analysed, 9.9% of patients received a test not needed according to the guidelines. There was an increase in recommended tests that were not performed per patient of 0.05 ± 0.27 in the control group compared to 0.55 ± 1.00 in the intervention group (*P* < .001); 4.2% of control patients missed at least one necessary test compared to 30.1% in the intervention group. Nine percent of intervention patients had a necessary test omitted. Guideline adherence (correctly tested/not tested) improved for all tests, with 1.6% in the control group compared to 49.3% in the intervention group (*P* < .001). Cost savings equated to Euro 34.66 per patient.
Ong 2017, USA [50]	Radiology staff	Radiology issues and associated resolutions	Cost to develop the tool. Number of issues filed for resolution. Radiologist-to-technologist feedback rate.	The tool was reported to be cost-effective and enables communication between radiologists and technologists at the point of patient care to provide feedback on quality issues.
Dunscombe 2015, USA [24]	Clinical staff in radiation oncology	User experience and participation	Feedback questionnaire which ascertained ease of use, intention to use again, opinion about the ability of the tool to improve safety, whether collaborative use enhanced communication, and whether multidisciplinary use enhanced the safety culture of the department.	The tool was found to be practical and efficient, and allows for benchmarking against comparable organizations. The downloadable log can facilitate QI over time. User experience was generally favourable during the piloting of the tool.
Hauschildt 2017, USA [51]	Clinical ethics consultants	Experience with the online comment system approach to clinical ethics consultation	Analysis of consultation logs (case summaries, recommendations, and comments) to assess how system was used and the extent that it achieved goals of consensus, quality assurance and education.	The online ethics comment system facilitated broad committee participation and consensus building. The electronic medium allows for meaningful discussion and deliberation of cases and recommendations. The process assists in improving the quality of ethics consultations, ensuring that relevant details are considered and that the recommendations made are based on input from committee members.
Quigley 2019, USA [33]	Nursing home staff	Ability to support quality nursing home care	Usability, accessibility, and prominence. Website design (DISCERN measure). Availability of training materials. Recency of update. Identification of key personnel. Quality focus areas.	The websites differed in ease of accessing the information. Over half of the websites did not provide customized resources, offering the same information for all states. Two websites displayed topics on the landing page, six displayed the information in another prominent way and the remaining two did not. The other six websites commonly provided access to resources on antibiotic stewardship and the National Healthcare Safety Network. Websites also provided information and factsheets on avoiding hospitalization. Resources (e.g. tool kits, webinars, training, and contact information) on reducing avoidable hospitalizations were available to 23 states. There was varied access to resources on infection control that were available to 34 states. Eleven websites did not include an update date.
Babul 2020, Canada [52]	Emergency and GP family physicians	General uptake of the CATT tool, and quality assurance/quality improvement assessment	Uptake of tool measured through Google Analytics. Quality Assurance/Quality Improvement measured through survey.	Google Analytic results: In Year 1, there were 8072 pageviews, and in Year 2, 9382 pageviews; 89 participants completed the Quality Assurance/Quality Improvement survey; 85% stated that they learnt new information, 73% stated that they made changes to the way that they diagnose, treat or manage concussion, 71% stated that they recommended the tool to other health professionals, 45% stated that they accessed other tools from the CATT website, and 48% stated that they had used CATT patient resources.
Fossouo Tagne 2023, Australia [30]	Community pharmacists	Usefulness and satisfaction of the GuildCare Web-based Adverse Drug Reaction Recording System	Semi-structured interview, think-aloud moderating techniques, retrospective questioning to evaluate usefulness and satisfaction. System Usability Scale to evaluate satisfaction. Remote observation was used to evaluate usability.	System Usability Score of 68.57 (above average). Functional and user interpretation issues were identified, such as unnecessary information, lack of system clarity, and redundant data fields. These insights were gained from the interview protocol, not the System Usability Score. Design elements like drop-down menus, free-text entry, checkboxes, and prefilled or auto-populated data fields were perceived as useful for enhancing system navigation and facilitating adverse drug reaction reporting.
Yi 2023, USA [53]	Emergency clinicians	Assess usability	Quantitative survey, System Usability Scale (SUS) and Net Promotor Score (NPS).	The results reflected overall ease of use with need for only minor improvements (SUS score of 73.2; minimum 47.5, maximum 92.5). A SUS score >68 is considered above average in overall measure of system satisfaction and sub-scales of usability and learnability. Respondents reported that they are likely to refer colleagues to the Dashboard (NPS score 20.7). All but 1 respondent learned something new from the dashboard, and 77% felt that the dashboard enhanced their understanding of patients’ emergency department experiences.

### Study quality

Risk of bias results are outlined in Supplementary File 3. Most studies (*n* = 16) had clear research objectives and appropriate methods. Four studies described tool development only and lacked formal data analyses. While most qualitative studies justified their approach, three studies lacked detail regarding the methodology. Assessing some quantitative studies were difficult due to lack of detailed methodology, sampling strategy, or measurements.

### Narrative synthesis

There were three main themes that emerged: tool development processes, QI mechanisms, and barriers and facilitators to tool usage. Overall synthesis quality, as per the GRADE framework [18], was low due to the incorporation of qualitative studies.

### Tool development processes

Eleven studies described the tool development process. Four studies undertook research or literature review prior to developing the tool, one study utilzsed a theoretical framework to guide tool development, nine studies co-developed the tool or consulted with stakeholders in the development process and four studies conducted field testing.

The most adopted process was co-developing with stakeholders. The approaches varied across studies, reflecting different levels of stakeholder engagement and adoption of co-design methodologies. For instance, two studies formed specialized working groups, emphasizing collaboration among healthcare practitioners with expertise in the relevant fields [19, 21]. Another study involved cyclical feedback from various stakeholders, including healthcare associations and quality improvement bodies, reflecting a broader engagement strategy [23]. Two studies demonstrated collaboration between professional societies and advisory groups to develop tools tailored to specific clinical domains [22, 24].

The least commonly adopted process was utilizing a theoretical framework (*n* = 1). The development of the ‘Cancer Care Quality Improvement Toolkit’ [25] was guided by Roger’s Diffusion of Innovation Model [26], and analyses were guided by the Consolidated Framework for Implementation Research [27].

### QI mechanisms

Table 3 summarizes the primary QI process/mechanisms that the tools utilized. These included digitalizing current QI processes (transforming traditional, paper-based, or manual methods into digital formats) (*n* = 7), identifying gaps in practice (*n* = 6), professional development (*n* = 3), and using clinical governance and organizational management as part of QI (*n* = 2). Some studies described the benefits of these processes, for example, digitalization was found to be feasible and preferred over paper-based methods [28, 29]. These processes supported healthcare professionals by enhancing accuracy and efficiency in clinical tasks, improving adherence to guidelines, facilitating reflective practice, and providing tailored feedback for continuous quality improvement. For example, the *EQUSUM* tool [28] for endometriosis surgery significantly improved classification accuracy and usability compared to paper methods.

**Table 3.** Website-based tools grouped by primary QI process/mechanism.

Primary QI process/mechanism	Identified tools
Identify gaps in practice	The endoscopy Global Rating Scale-Canada (GRS-C) [19] Global Rating Scale for Inflammatory Bowel Disease [21] The Residency Performance Index (RPI) [20] Online self-audit of methadone treatment in Irish general practice [31] Safety Profile Assessment (SPA) [24, 54] Paediatric Endoscopy Global Rating Scale [22]
Use clinical governance and organizational management as part of quality improvement	Primary Care Practice Improvement Tool (PC-PIT) [23] Digital equity dashboard for the emergency department [53]
Digitalize current QI processes	Endometriosis QUality and grading instrument for SUrgical performance (EQUSUM) [28] Online Toolkit for Measuring Performance in Health Emergency Response Exercises [49] Pastoral Care Activity Tracker (PCAT) [29] Electronic decision support tool ‘PReOPerative evaluation’ (PROP) [32] Image Quality Reporting and Tracking Solution (IQuaRTS) [50] Online comment system in clinical ethics consultation [51] Web-Based Tool to Report Adverse Drug Reaction [30]
Professional development	[55]Web-Based Cancer Care Quality Improvement Toolkit [25] Quality Innovation Networks (QIN) [33] The Concussion Awareness Training Tool (CATT) [52]

There were a variety of QI features. The most common were education/training resources (*n* = 7), assisting the user to assess current practices against standards or recommendations (*n* = 6), and recording activity or performance (*n* = 6). For example, the ‘Safety Profile Assessment’ [24] helped assess compliance with safety/quality indicators in radiation therapy using a 5-point Likert scale. Other features reported were an automated calculation/scoring system (*n* = 4), such as the ‘Paediatric Endoscopy Global Rating Scale’ [22] where each measure was assigned a level from D to A and a score was generated for each standard. In addition, action plans or recommendations for QI (*n* = 3), downloadable forms (*n* = 4), self-assessment of current practices (*n* = 2), and platforms for sharing data or information (*n* = 4) were reported.

Some studies elaborated on how the tool’s features were advantageous in QI and resulted in better clinical outcomes, increased professional satisfaction, and streamlined workflow. The ‘Web-Based Tool to Report Adverse Drug Reactions’ [30] allowed for recording of adverse events with all the necessary information for submission to the Australian Therapeutic Goods Administration, in line with professional requirements. The ‘self-audit of methadone treatment’ [31] tool could analyse the inputted data and provide a comparison to the expected standards.

### Barriers and facilitators

Thirteen studies reported barriers and facilitators to tool usage (Table 4). There were three main factors contributing to the facilitators in tool usage.

**Table 4.** Analysis of facilitators and barriers to tool usage.

Main factors identified	Subtopic	Extracts
Facilitators		
Relevance to practice	Usefulness to practice Alignment with professional standards/requirements	‘Usefulness of tool during annual program evaluation’ [20] ‘Relevance to everyday practice work and planning’ [23] ‘Reminder of importance of standards and key protocols’ [31] ‘Alignment with emergency response capabilities and relevant to goals of organization’ [49] ‘Advice and experience from a variety of authoritative sources that allows benchmarking against comparable institutions’ [24]
Accessibility	Easy to use User-friendly Enhances current practices	‘Easy and preferable to complete online than paper-based form’ [23] ‘Visual advantages with anatomical pictures for classification’ [28] ‘Easy to complete’ [31] ‘Automatic reporting’ [29] ‘User friendly’ [32] ‘Easy to navigate’ [33, 55] ‘Ability to customize evaluation form’ [49] ‘Direct submission of the report to the Therapeutic Goods Administration’ [30]
Facilitating multidisciplinary action	Allows for shared decision-making Increasing engagement with stakeholders	‘Allowing staff to be involved in identification of areas for improvement’ [23] ‘Allows for interdepartmental coordination’ [28] ‘Increasing engagement with surgical colleagues on delivery of endoscopy services’ [22]
Barriers		
Time consuming	Using the tool took up too much time or effort Resources were too long	‘The time required’ [20, 55] ‘Lengthy and protracted’ [31] ‘Arduous (lots of effort)’ [29]
Irrelevant to practice	Information not specific to practice Technical concerns with implementation	‘Covered areas that may be outside clinical management processes’ [23] ‘Unable to be integrated into electronic health records used in outpatient sector in Australia’ [32] ‘Concern with ability of tool to facilitate quality and safety improvement’ [24]
Difficult to use	Content is difficult to understand Technical difficulties for the user	‘Elements difficult to understand’ [23] ‘Poor clarity of questions’ [31] ‘Not user-friendly or easy to navigate’ [33]
Lack of organization engagement	Organization barriers to adopting new tool Lack of organization support required for implementation	‘Reluctance to change’ [25] ‘Required approvals’ [25] ‘Resource constraints and clinical pressures’ [24] ‘Low staffing… and the need for trust and managerial support’ [22]

Relevance to practice: the tool was considered useful and aligned with professional standards (*n* = 5). For example, General Practice staffs who used the ‘self-audit of methadone treatment’ [31] found that the tool was helpful in reflection of their clinical practice and served as a reminder of the importance of standards. The staff that used the ‘Primary Care Practice Improvement Tool (PC-PIT)’ [23] thought that the tool was relevant to everyday practice work and planning, particularly as the tool addressed clinical governance.

Accessibility: the tool was user-friendly and enhanced current practices through a web-based interactive platform. For example, endometriosis experts who utilized the *EQUSUM* tool [28] found that the tool was easy to use and there were visual advantages of using anatomical pictures for classification.

Facilitating multidisciplinary action: the tool allowed for shared decision-making and increased engagement with stakeholders. For example, one of the identified facilitators of the ‘PC-PIT’ tool [23] was that it allowed all staff to be involved in the identification of areas for improvement. The ‘Paediatric Endoscopy Global Rating Scale’ [22] found that the tool increased engagement with surgical colleagues on delivery of endoscopy services.

There were four main factors contributing to the barriers in tool usage.

Time consuming: the tool took users too long to use. For example, some users found the ‘Residency Performance Index’ [20] tool time-consuming to use and the ‘self-audit of methadone treatment’ [31] tool lengthy and protracted.

Irrelevant to practice: the tool did not provide information specific to practice, or there were concerns with implementation in the real-life setting. The ‘PC-PIT’ tool [23] covered areas that some users thought may be outside clinical management processes. The ‘PROP’ tool [32] study identified a technical barrier where the tool was unable to be integrated into electronic health records.

Difficult to use: the information offered in the tool was difficult to understand, or technical difficulties were experienced with using the tool. The ‘PC-PIT’ tool [23] contained elements that were difficult to understand for some users. The ‘self-audit of methadone treatment’ [31] had poor clarity of questions for some, and ‘Quality Innovation Networks’ [33] was not considered easy to navigate or use by some.

Lack of organization engagement: there were organization barriers that prevented implementation of the tool. The ‘Cancer Care Quality Improvement Toolkit’ [25] study identified that there was some reluctance to change and the tool required approvals for implementation that prevented wider use. The ‘Safety Profile Assessment’ tool [24] study found that resource constraints and clinical pressures prevented tool usage.

## DISCUSSION

### Statement of principal findings

The findings highlight that identifying gaps in practice, digitalizing existing processes and contributing to professional development were key mechanisms that tools adopted. These tools were associated with reported enhancement of efficiency in clinical tasks, improvement in adherence to guidelines, facilitation of reflective practice, and provision of tailored feedback for continuous quality improvement. The most common tool features were education/training resources and the ability to assist the user in assessing current practices, which supported healthcare professionals in achieving better clinical outcomes, increased professional satisfaction and streamlined workflow in various healthcare settings. Reported facilitators to tool usage included relevance to practice, accessibility, and facilitating multidisciplinary action, making these tools practical and time-efficient for healthcare settings. Barriers reported included being time-consuming, irrelevant to practice, difficult to use, and lack of organizational engagement, highlighting areas for improvement. The co-design approaches varied across studies, reflecting different levels of stakeholder engagement and adoption of co-design methodologies.

### Strengths and limitations

This study adhered to the PRISMA statement and followed the registered study protocol in PROSPERO. This was the first systematic review to summarize how website-based tools can support health professionals with quality improvement. However, there are several limitations. Inclusion of qualitative studies prevented the ability to perform data analyses and overall quality for syntheses was low. Heterogeneous study designs, outcomes, and methodologies limited generalizability of findings. Some studies lacked the details regarding methodology, including justification for chosen designs. Narrative reviews may have limitations in terms of objectivity.

### Interpretation within the context of the wider literature

Many tools in the current review helped the user to identify gaps in practice and this was suggested to be useful in QI, with ‘relevance to practice’ identified as a facilitator to tool usage. Identifying gaps in practice is a common feature across many QI projects in professional settings. For example, an auditing tool for surgical QI was designed to assess compliance with infection-related process measures and identify gaps in measure implementation. It was found that the tool was useful in identifying gaps and quarterly compliance improved for 80% of process measures [34]. Another tool for assessing resident’s competence was developed to provide data on gaps in knowledge, which could be used to guide curriculum development [35]. Identifying the gaps, especially between evidence and practice or policy-making, is one of the crucial first steps in knowledge translation [36]. This is likely why tools that can identify gaps in practice prove to be valuable in QI initiatives and can be used during the formative planning process.

The findings of the current review show that many website-based tools have been developed to digitalize existing QI processes, and ‘accessibility’ was identified as a facilitator to tool usage. A study examining digital tools for patient monitoring in oncology care found that digitalization allowed for nurse practitioners to efficiently extend and improve symptom management [37]. Another study found that a digital tool for a home-based blood pressure monitoring programme was effective and timely in hypertension management [38]. In recent years, digital transformation has been observed across many professional sectors and this has been associated with numerous advantages. For instance, the implementation of the Electronic Health Record has led to improvements in quality of care, reduction in medical errors, and increased adherence to clinical guidelines [39]. Considering how important QI is in the workforce, it was not surprising that many tools analysed in the current review sought to digitalize existing processes.

Although digitalization has many advantages, there are limitations that should be considered. In the current review, a barrier to tool usage was the tool being time consuming or difficult to use. Similarly, a systematic review examining web-based interventions for weight loss highlighted that one of the challenges was engagement and retention. The authors suggested that there is a need to investigate components of web-based tools that can maintain users’ motivation and interest [40]. A study exploring digital tools to deliver physical activity advice identified that the biggest influence was having the skills to utilize the tool [41]. These observations, including the findings of the current review, suggest that digital literacy is an important consideration when developing digital tools or interventions and addressing these barriers may assist in maximizing user engagement and successful tool implementation.

Another key QI process that the tools in the current review adopted was contributions to professional development. Education/training resources were also identified as one the common tool features. Many QI tools are designed to assist with professional development. For example, a digital application designed for teachers to improve their students’ linguistic competence assisted the teacher to self-assess their classes, make decisions, and facilitate practice changes through professional development. The use of the tool led to improved competencies for both the teachers and students [42]. A study that reported the development of a self-assessment tool for dental faculty to map professional growth identified the lack of defined faculty competencies in medical and dental education. Subsequently, a tool was developed and could be used as part of professional development [43]. Literature across all professions have demonstrated great benefits in undertaking professional development and this highlights why it is an ongoing requirement of practice for some. For example, continuing professional development is a requirement for nurses and has been directly linked to nurses’ career satisfaction and continuous growth in their practice [44].

In the current review, almost all tools were co-developed with stakeholders. This finding is further supported by ‘facilitating multidisciplinary action’ being identified as a facilitator of tool usage. Similarly, a QI project to improve ambulatory care for patients with musculoskeletal disorders focused on collaboration between general practitioners and specialists [45]. The study found that collaborative care was associated with a lower risk of osteoarthritis-related hospitalization, higher participation in exercise interventions, and more frequently prescribed physical therapy. The benefits of collaboration can be attributed to many factors, such as the opportunity to learn from others, access to new resources, increased productivity, and shared goals [46].

The current review identified lack of organizational engagement as a barrier, which included reluctance to change. A study exploring resistance towards changes among healthcare staff identified several reasons for reluctance, including personal reluctance, misunderstanding of project/initiative aims, and a dislike of the methods by which projects have been promoted [47]. A QI initiative for educational programmes found that there was reluctance among some teachers due to their uncertainty about potential benefits that could arise from the initiative [48]. In the current review, the qualitative data indicated factors, such as resource constraints, low staffing, and managerial support as barriers. These findings indicate that the reasons for lack of organizational engagement are multifactorial, and further research exploring this barrier would be beneficial.

### Implications for policy, practice, and research

The findings offer valuable insights for future development of QI website-based tools. It is expected that more digital tools will be developed to drive practice improvements and the findings of this review can be useful in guiding the development process. Recommendations include ensuring tools are co-developed with healthcare professionals, focusing on practical usability, and addressing common barriers to enhance engagement and effectiveness in improving healthcare quality. The insights into the barriers and facilitators of tool usage can be broadly applied to any project that seeks to improve practice. Randomized controlled trials are warranted to provide objective evidence of tool efficacy.

## Conclusion

The findings emphasize the importance of co-development with healthcare professionals, practical usability, and addressing barriers to enhance engagement and effectiveness of QI digital tools. There is a lack of randomized controlled trials on the efficacy of these tools. Future work should address this knowledge gap.

## Supplementary Material

mzae068_Supp

## Data Availability

No new data were generated or analysed in support of this research.
