# Supplementary material for: The utility of website-based quality improvement tools for health professionals: a systematic review
Source: Int J Qual Health Care. 2024 Jul 10;36(3):mzae068. doi: 10.1093/intqhc/mzae068 (PMC11277856; doi:10.1093/intqhc/mzae068)
Supplement: mzae068_Supp [file mzae068_supp.zip › suppl_data/Supplementary file 2.docx]

**Supplementary file 2.** Search strategy and terms for each database

Published data January 2012 to January 2024

Language: English

Document type: article

| **Database** | **Search terms** | ***n* (articles)** |
| --- | --- | --- |
| PubMed | Search ((("quality improvement"[MeSH Terms] OR ("quality"[All Fields] AND "improvement"[All Fields]) OR "quality improvement"[All Fields] OR (("qualities"[All Fields] OR "quality"[All Fields] OR "quality s"[All Fields]) AND "tool"[All Fields])) AND "web-based"[All Fields]) OR "website-based"[All Fields]) | 3509 |
| MEDLINE | S1. quality improvement* OR quality improvement tool OR quality tool OR qualities OR quality  S2. web-based OR website-based  S3. Combine S1 and S2 with “AND” | 764 |
| ScienceDirect | (quality improvement tool) OR (quality tool) AND (web-based) OR (website-based) | 397 |
| Wiley | [All: quality improvement] AND [[All: tool] OR [All: quality]] AND [All: tool] AND [All: web-based] OR [All: website-based] | 160 |
| Scopus | S1. quality improvement* OR quality improvement tool OR quality tool OR qualities OR quality  S2. web-based OR website-based  S3. Combine S1 and S2 with “AND” | 220 |
| ProQuest | S1. quality improvement* OR quality improvement tool OR quality tool OR qualities OR quality  S2. web-based OR website-based | 163 |
| Education Resource Complete | S1. quality improvement* OR quality improvement tool OR quality tool OR qualities OR quality  S2. web-based OR website-based | 58 |
| A+ Education | S1. quality improvement* OR quality improvement tool OR quality tool OR qualities OR quality  S2. web-based OR website-based | 37 |
| **Total** | | **5308** |
