# Supplementary material for: The utility of website-based quality improvement tools for health professionals: a systematic review
Source: Int J Qual Health Care. 2024 Jul 10;36(3):mzae068. doi: 10.1093/intqhc/mzae068 (PMC11277856; doi:10.1093/intqhc/mzae068)
Supplement: mzae068_Supp [file mzae068_supp.zip › suppl_data/Supplementary file 3.docx]

**Supplementary file 3.** Risk of bias assessments for each included study

**Table 1.** Studies assessed using Mixed Methods Appraisal Tool (MMAT)

| Study | Are there clear research questions? | Do the collected data allow to address the research questions? | Is the qualitative approach appropriate to answer the research question? | Are the qualitative data collection methods adequate to address the research question? | Are the findings adequately derived from the data? | Is the interpretation of results sufficiently substantiated by data? | Is there coherence between qualitative data sources, collection, analysis and interpretation? |
| --- | --- | --- | --- | --- | --- | --- | --- |
| Qualitative studies | | | | | | | |
| MacIntosh 2013 [1] | Y | CT | Y | Y | Y | CT | Y |
| Hoekzema 2014 [2] | Y | CT | Y | CT | Y | CT | N |
| Van Hout 2018 [3] | Y | Y | Y | Y | Y | Y | Y |
| Agboola 2015 [4] | Y | Y | Y | Y | Y | CT | Y |
| Bowman 2015 [5] | Y | Y | Y | Y | Y | Y | Y |
| Hauschildt 2017 [6] | N | Y | CT | CT | Y | Y | CT |
| Quigley 2019 [7] | Y | Y | Y | Y | Y | CT | Y |
| Babul 2020 [8] | Y | Y | Y | CT | Y | CT | Y |
| Narula 2019 [9] | Y | Y | Y | Y | Y | N | CT |
| Non-randomised studies | | | | | | | |
| Study | **Are there clear research questions?** | **Do the collected data allow to address the research questions?** | **Are the participants representative of the target population?** | **Are measurements appropriate regarding both the outcome and intervention (or exposure)?** | **Are there complete outcome data?** | **Are the confounders accounted for in the design and analysis?** | **During the study period, is the intervention administered (or exposure occurred) as intended?** |
| Flamm 2013 [10] | Y | Y | Y | Y | Y | CT | Y |
| Quantitative descriptive studies | | | | | | | |
| Study | **Are there clear research questions?** | **Do the collected data allow to address the research questions?** | **Is the sampling strategy relevant to address the research question?** | **Is the sample representative of the target population?** | **Are the measurements appropriate?** | **Is the risk of nonresponse bias low?** | **Is the statistical analysis appropriate to answer the research question?** |
| Bitton 2020 [11] | Y | Y | CT | CT | Y | Y | Y |
| Calder 2022 [12] | Y | Y | Y | Y | Y | N | Y |
| Dunscombe 2015 [13] | CT | CT | CT | Y | Y | N | Y |
| Metzemaekers 2020 [14] | Y | Y | CT | CT | CT | CT | CT |
| Ong 2017 [15] | Y | CT | CT | N | Y | CT | Y |
| Mixed method studies | | | | | | | |
| Study | **Are there clear research questions?** | **Do the collected data allow to address the research questions?** | **Is there an adequate rationale for using a mixed methods design to address the research question?** | **Are the different components of the study effectively integrated to answer the research question?** | **Are the outputs of the integration of qualitative and quantitative components adequately interpreted?** | **Are divergences and inconsistencies between quantitative and qualitative results adequately addressed?** | **Do the different components of the study adhere to the quality criteria of each tradition of the methods involved?** |
| Crossland 2014 [16] | Y | Y | Y | Y | CT | CT | Y |
| Fossouo Tagne 2023 [17] | Y | Y | Y | Y | Y | CT | Y |
| Yi 2023 [18] | Y | Y | CT | CT | Y | CT | Y |

Y: Yes

N: No

CT: Can’t Tell

**References**

[1] D. MacIntosh, C. Dubé, R. Hollingworth, S. V. van Zanten, S. Daniels, and G. Ghattas, “The Endoscopy Global Rating Scale – Canada: Development And Implementation of a Quality Improvement Tool,” *Can. J. Gastroenterol. Hepatol.*, vol. 27, no. 2, pp. 74–82, 2013, doi: 10.1155/2013/165804.

[2] G. S. Hoekzema, L. Maxwell, J. W. G. Jr, W. W. Mills, and W. Geiger, “The Residency Performance Index: An Effort at Residency Quality Assessment and Improvement in Family Medicine,” *J. Grad. Méd. Educ.*, vol. 6, no. 4, pp. 756–759, 2014, doi: 10.4300/jgme-d-13-00355.1.

[3] M. C. V. Hout, D. Crowley, A. McBride, and I. Delargy, “Piloting online self-audit of methadone treatment in Irish general practice: results, reflections and educational outcomes,” *BMC Méd. Educ.*, vol. 18, no. 1, p. 153, 2018, doi: 10.1186/s12909-018-1259-2.

[4] F. Agboola, D. Bernard, E. Savoia, and P. D. Biddinger, “Development of an Online Toolkit for Measuring Performance in Health Emergency Response Exercises,” *Prehospital Disaster Med.*, vol. 30, no. 5, pp. 503–508, 2015, doi: 10.1017/s1049023x15005117.

[5] C. Bowman, J. Luck, R. C. Gale, N. Smith, L. S. York, and S. Asch, “A Qualitative Evaluation of Web-Based Cancer Care Quality Improvement Toolkit Use in the Veterans Health Administration,” *Qual. Manag. Heal. Care*, vol. 24, no. 3, pp. 147–161, 2015, doi: 10.1097/qmh.0000000000000063.

[6] K. Hauschildt, T. K. Paul, R. D. Vries, L. B. Smith, C. J. Vercler, and A. G. Shuman, “The use of an online comment system in clinical ethics consultation,” *AJOB Empir. Bioeth.*, vol. 8, no. 3, pp. 153–160, 2017, doi: 10.1080/23294515.2017.1335808.

[7] D. D. Quigley, A. Dick, and P. W. Stone, “Quality Innovation Networks Share Varied Resources for Nursing Homes on Mostly User‐Friendly Websites,” *J. Am. Geriatr. Soc.*, vol. 67, no. 11, pp. 2376–2381, 2019, doi: 10.1111/jgs.16201.

[8] S. Babul, K. Turcotte, M. Lambert, G. Hadly, and K. Sadler, “Delivering Evidence-Based Online Concussion Education to Medical and Healthcare Professionals: The Concussion Awareness Training Tool (CATT),” *J. Sports Med.*, vol. 2020, p. 8896601, 2020, doi: 10.1155/2020/8896601.

[9] P. Narula *et al.*, “Paediatric Endoscopy Global Rating Scale,” *J. Pediatr. Gastroenterol. Nutr.*, vol. 69, no. 2, pp. 171–175, 2019, doi: 10.1097/mpg.0000000000002355.

[10] M. Flamm *et al.*, “Quality improvement in preoperative assessment by implementation of an electronic decision support tool,” *J. Am. Méd. Inform. Assoc.*, vol. 20, no. e1, pp. e91–e96, 2013, doi: 10.1136/amiajnl-2012-001178.

[11] A. Bitton *et al.*, “Development of a Global Rating Scale for Inflammatory Bowel Disease,” *J. Can. Assoc. Gastroenterol.*, vol. 3, no. 1, pp. 4–16, 2020, doi: 10.1093/jcag/gwz017.

[12] S. Calder, M. Andreotta, T. Morris, and M. Atee, “Improving quality in pastoral care using the Pastoral Care Activity Tracker (PCAT): A feasibility study of a digital tool within an Australian healthcare organization,” *J. Heal. Care Chaplain.*, vol. 29, pp. 353–367, 2023, doi: 10.1080/08854726.2022.2091837.

[13] P. Dunscombe *et al.*, “Safety Profile Assessment: An online tool to gauge safety-critical performance in radiation oncology,” *Pr. Radiat. Oncol.*, vol. 5, no. 2, pp. 127–134, 2015, doi: 10.1016/j.prro.2014.10.012.

[14] J. Metzemaekers *et al.*, “EQUSUM: Endometriosis QUality and grading instrument for SUrgical performance: proof of concept study for automatic digital registration and classification scoring for r-ASRM, EFI and Enzian,” *Hum. Reprod. Open*, vol. 2020, no. 4, pp. hoaa053-, 2020, doi: 10.1093/hropen/hoaa053.

[15] L. Ong, P. Elnajjar, C. G. Nyman, T. Mair, and K. Juluru, “Implementation of a Point-of-Care Radiologist-Technologist Communication Tool in a Quality Assurance Program,” *Am. J. Roentgenol.*, vol. 209, no. 1, pp. W18–W25, 2017, doi: 10.2214/ajr.16.17517.

[16] L. Crossland, T. Janamian, M. Sheehan, V. Siskind, J. Hepworth, and C. L. Jackson, “Development and pilot study of the Primary Care Practice Improvement Tool (PC‐PIT): an innovative approach,” *Méd. J. Aust.*, vol. 201, no. S3, pp. S52–S55, 2014, doi: 10.5694/mja14.00262.

[17] J. F. Tagne, R. A. Yakob, R. Mcdonald, and N. Wickramasinghe, “A Web-Based Tool to Report Adverse Drug Reactions by Community Pharmacists in Australia: Usability Testing Study,” *JMIR Form. Res.*, vol. 7, p. e48976, 2023, doi: 10.2196/48976.

[18] S. Yi, C. Burke, A. Reilly, S. Straube, J. Graterol, and C. R. Peabody, “Designing and developing a digital equity dashboard for the emergency department,” *J. Am. Coll. Emerg. Physicians Open*, vol. 4, no. 4, p. e12997, 2023, doi: 10.1002/emp2.12997.
